# Drug targeting to decrease cardiotoxicity – determination of the cytotoxic effect of GnRH-based conjugates containing doxorubicin, daunorubicin and methotrexate on human cardiomyocytes and endothelial cells

**DOI:** 10.3762/bjoc.14.136

**Published:** 2018-06-28

**Authors:** Livia Polgár, Eszter Lajkó, Pál Soós, Orsolya Láng, Marilena Manea, Béla Merkely, Gábor Mező, László Kőhidai

**Affiliations:** 1 Heart and Vascular Center, Semmelweis University, Városmajor u. 68., Budapest, 1122, Hungary; 2 Chemotaxis Research Group, Department of Genetics, Cell- and Immunobiology, Semmelweis University, Nagyvárad tér 4., Budapest, 1089, Hungary; 3 University of Konstanz, Department of Chemistry and Zukunftskolleg, Universitätsstrasse 10, 78467 Konstanz, Germany; 4 Eötvös Loránd University, Faculty of Science, Institute of Chemistry, Pázmány P. stny 1/A Budapest, 1117, Hungary; 5 MTA-ELTE Research Group of Peptide Chemistry, Pázmány P. stny 1/A, Hungarian Academy of Science, Budapest, 1117, Hungary

**Keywords:** cardiotoxicity, drug targeting, GnRH-conjugates, HCM, HUVEC, impedimetry

## Abstract

**Background:** Cardiomyopathy induced by the chemotherapeutic agents doxorubicin and daunorubicin is a major limiting factor for their application in cancer therapy. Chemotactic drug targeting potentially increases the tumor selectivity of drugs and decreases their cardiotoxicity. Increased expression of gonadotropin-releasing hormone (GnRH) receptors on the surface of tumor cells has been reported. Thus, the attachment of the aforementioned chemotherapeutic drugs to GnRH-based peptides may result in compounds with increased therapeutic efficacy. The objective of the present study was to examine the cytotoxic effect of anticancer drug–GnRH-conjugates against two essential cardiovascular cell types, such as cardiomyocytes and endothelial cells. Sixteen different previously developed GnRH-conjugates containing doxorubicin, daunorubicin and methotrexate were investigated in this study. Their cytotoxicity was determined on primary human cardiac myocytes (HCM) and human umbilical vein endothelial cells (HUVEC) using the xCELLigence SP system, which measures impedance changes caused by adhering cells on golden electrode arrays placed at the bottom of the wells. Slopes of impedance–time curves were calculated and for the quantitative determination of cytotoxicity, the difference to the control was analysed.

**Results:** Doxorubicin and daunorubicin exhibited a cytotoxic effect on both cell types, at the highest concentrations tested. Doxorubicin-based conjugates (AN-152, GnRH-III(Dox-*O*-glut), GnRH-III(Dox-glut-GFLG) and GnRH-III(Dox=Aoa-GFLG) showed the same cytotoxic effect on cardiomyocytes. Among the daunorubicin-based conjugates, [^4^Lys(Ac)]-GnRH-III(Dau=Aoa), GnRH-III(Dau=Aoa-YRRL), {GnRH-III(Dau=Aoa-YRRL-C)}_2_ and {[^4^*N*-MeSer]-GnRH-III(Dau-C)}_2_ had a significant but decreased cytotoxic effect, while the other conjugates – GnRH-III(Dau=Aoa), GnRH-III(Dau=Aoa-K(Dau=Aoa)), [^4^Lys(Dau=Aoa)]-GnRH-III(Dau=Aoa), GnRH-III(Dau=Aoa-GFLG), {GnRH-III(Dau-C)}_2_ and [^4^*N*-MeSer]-GnRH-III(Dau=Aoa) – exerted no cytotoxic effect on cardiomyocytes. Mixed conjugates containing methotrexate and daunorubicin – GnRH-III(Mtx-K(Dau=Aoa)) and [^4^Lys(Mtx)]-GnRH-III(Dau=Aoa) – showed no cytotoxic effect on cardiomyocytes, as well.

**Conclusion:** Based on these results, anticancer drug–GnRH-based conjugates with no cytotoxic effect on cardiomyocytes were identified. In the future, these compounds could provide a more targeted antitumor therapy with no cardiotoxic adverse effects. Moreover, impedimetric cytotoxicity analysis could be a valuable technique to determine the effect of drugs on cardiomyocytes.

## Introduction

Gonadotropin-releasing hormone (GnRH) is a peptide hormone secreted by the hypothalamus, which stimulates the release of follicle-stimulating hormone (FSH) and luteinizing hormone (LH) from the pituitary. Thus, it represents the first step in the hypothalamus-pituitary-gonadal axis, which plays an important role in reproduction [1]. It has been reported that gonadotropin-releasing hormone receptors (GnRH-Rs) are highly expressed on the surface of tumor cells, especially in gynaecological malignant tumors (breast, ovarian and endometrial cancers) [2]. GnRH and its analogues (both agonists and antagonists) are used for the treatment of different types of cancer [3]. They can inhibit the tumor growth in a direct way, through the GnRH-Rs on tumor cells or by an indirect way, through the influence of hormone secretion by the pituitary [4]. GnRH-III (Glp-His-Trp-Ser-His-Trp-Lys-Pro-Gly-NH_2,_ where Glp is pyroglutamic acid) is a naturally occurring isoform of GnRH, which was first isolated from sea lamprey [5]. GnRH-III has been shown to exert an effective antitumor activity against a number of tumor types [6–8]. However, it exerted a significantly lower endocrine effect in mammals than the human GnRH (GnRH-I: Glp-His-Trp-Ser-Tyr-Gly-Leu-Arg-Pro-Gly-NH_2_) and other GnRH analogues [9]. This low hormonal effect might provide an advantage in the treatment of hormone-independent tumors [10].

Targeted drug delivery is a technique of high interest, by which cytotoxic drugs are attached to specific molecules (homing devices) with the aim of increasing the accumulation of the drug in the specific target cells. Consequently, this leads to a more efficient antitumor effect and to the reduction of potential adverse side effects. Small peptides that recognize target receptors on tumor cells might be suitable targeting moieties for this purpose. Hormone peptides, in particular, GnRH and somatostatin derivatives that possess antiproliferative effect on their own, are among the best candidates as homing peptides [10]. A.V. Schally and his co-workers developed the first GnRH derivative–drug conjugates for targeted tumor therapy. One of these compounds Zoptarelin doxorubicin (developmental code names AEZS-108, AN-152) Glp-His-Trp-Ser-Tyr-*D*-Lys(Dox-*O*-glut)-Leu-Arg-Pro-Gly-NH_2_ (where glut is glutaric acid) [11] reached phase III clinical trial, which was discontinued for all indications under development in May 2017 [12]. GnRH-III-based conjugates have been investigated in our laboratory as promising candidates for targeted drug delivery with positive results in human tumor cell lines, both related (e.g., MCF-7) and unrelated (e.g., HT-29, MonoMac6) to the reproductive system [13–15].

Doxorubicin, daunorubicin and methotrexate are clinically used chemotherapeutic agents, with applications in a variety of malignant tumorous diseases [16–17]. Doxorubicin and daunorubicin belong to the anthracycline family and act by damaging the DNA of the cancer cells. Methotrexate is an antimetabolite that inhibits the folate metabolism of tumor cells. All three drugs have a great number of adverse effects; doxorubicin and daunorubicin are especially known for their cardiotoxicity, leading to cardiomyopathy and heart failure [18–19]. These side effects can limit the applicability of these chemotherapeutic drugs. The conjugation of doxorubicin and daunorubicin to a GnRH-III-based targeting peptide could lead to decreased cardiotoxic effect through the more specific drug targeting.

Drug delivery systems containing doxorubicin, daunorubicin and methotrexate attached to various GnRH-III derivatives were previously designed, synthesized and characterized for their antitumor effects in our laboratories [14–15,20–25]. Several approaches including sequence modification of GnRH-III [20], incorporation of enzymatic cleavable oligopeptide spacers (e.g., GFLG, YRRL) [15,21], dimerization of the targeting GnRH-III unit [22], attachment of drugs through different linkages (e.g., ester, amide or oxime bond) [14,23], and multiplication of drugs [24–25] were pursued in order to achieve an increased antitumor effect.

The main objective of the study reported here was to evaluate the in vitro cardiotoxic effects (as undesired side effects) of sixteen GnRH-III-based conjugates containing doxorubicin, daunorubicin and methotrexate on two essential target cells of cardiovascular diseases: vascular endothelial cells and cardiomyocytes. The rate of cytotoxicity (antitumor effect) vs cardiotoxicity (toxic effects on cardiomyocytes and vascular endothelium) is an important factor for efficient drug development for targeted tumor therapy with minimized side effects. The technique used in this work – impedimetry – is a dedicated one for real-time monitoring of cells to distinguish short-term (0–2 hours) and long-term (0–72 hours) effects elicited by the drug or carrier–drug conjugates. The basic theory of impedimetry is that proliferation/viability of cells is well detectable by monitoring of the electric impedance (*Z*) in an AC environment, due to the electric insulator character of intact surface membranes composed of the phospholipid bilayer. Application of cytotoxic compounds results in disturbed electrophysical integrity (decreasing value of impedance) of surface membranes as a signal of cell death. While cell physiological responses of short-term exposures result in characteristic changes of cell morphology (e.g., spreading), long-term effects characteristically influence in particular the proliferation of cells, and therefore the cytotoxic effects. The presented dimensionless values of delta slope (DS) measure the difference between the slope of the curve corresponding to the treatment and that of the control curve in impedimetric recordings, the negative signs refer to the relative values to the identical control.

In the following, the long-term effects of GnRH-III-based conjugates are presented in detail (see Figure 3b–g and Figure 4b–g), whereas the data on short-term effects are presented in Supporting Information File 1. As mentioned above, the modified cytotoxic/cardiotoxic moiety of antitumor drugs in GnRH conjugates is focused in the present work.

## Results

### Synthesis of GnRH derivative–drug conjugates

**Figure 1:**
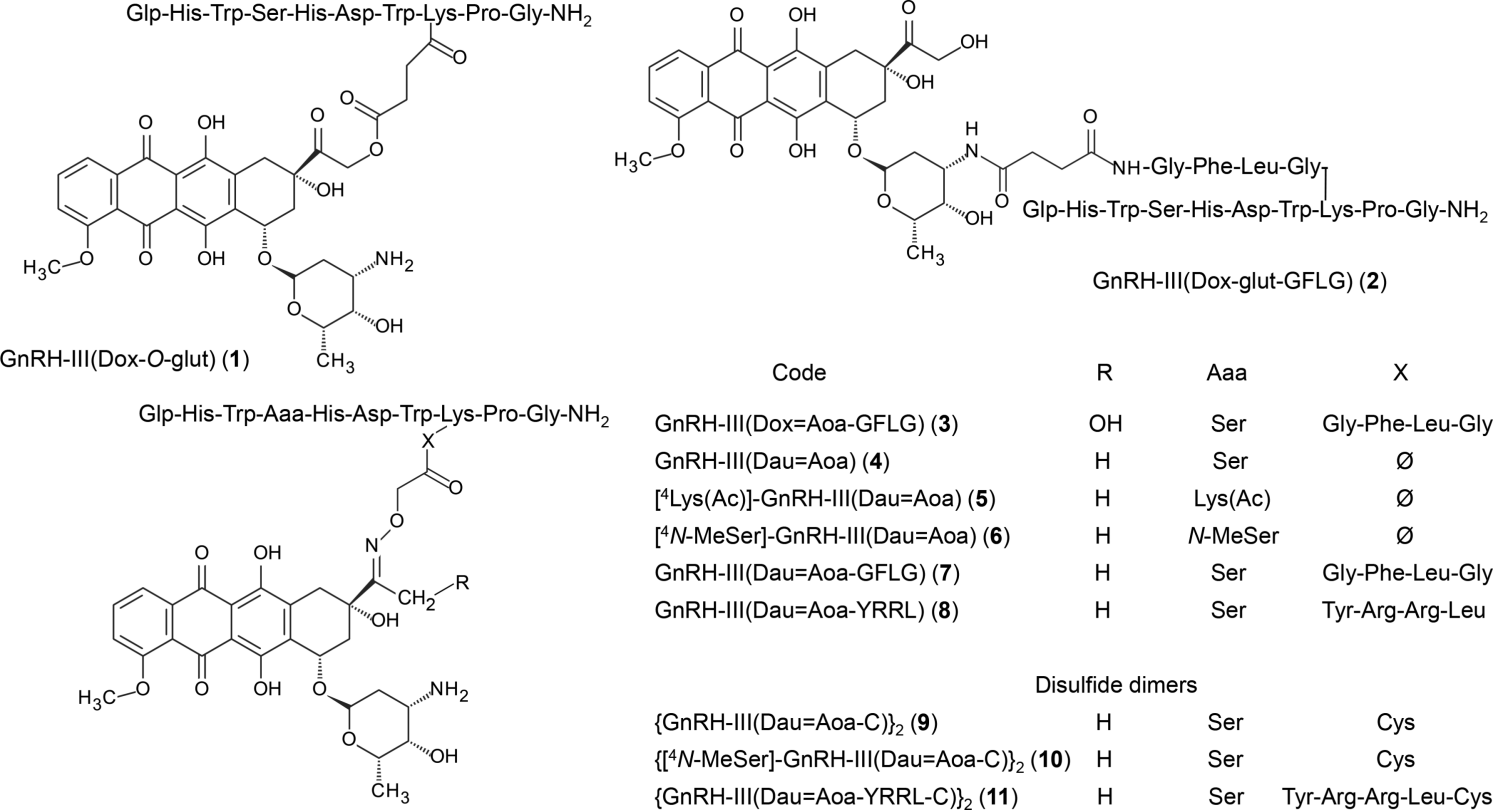
Chemical structures of the GnRH-drug conjugates synthesized and investigated in the present work. Abbreviations: Aoa: aminooxyacetyl; Dox: doxorubicin; Dau: daunorubicin; Mtx: methotrexate; Glp: pyroglutamic acid; gult: glutaric acid.

**Figure 2:**
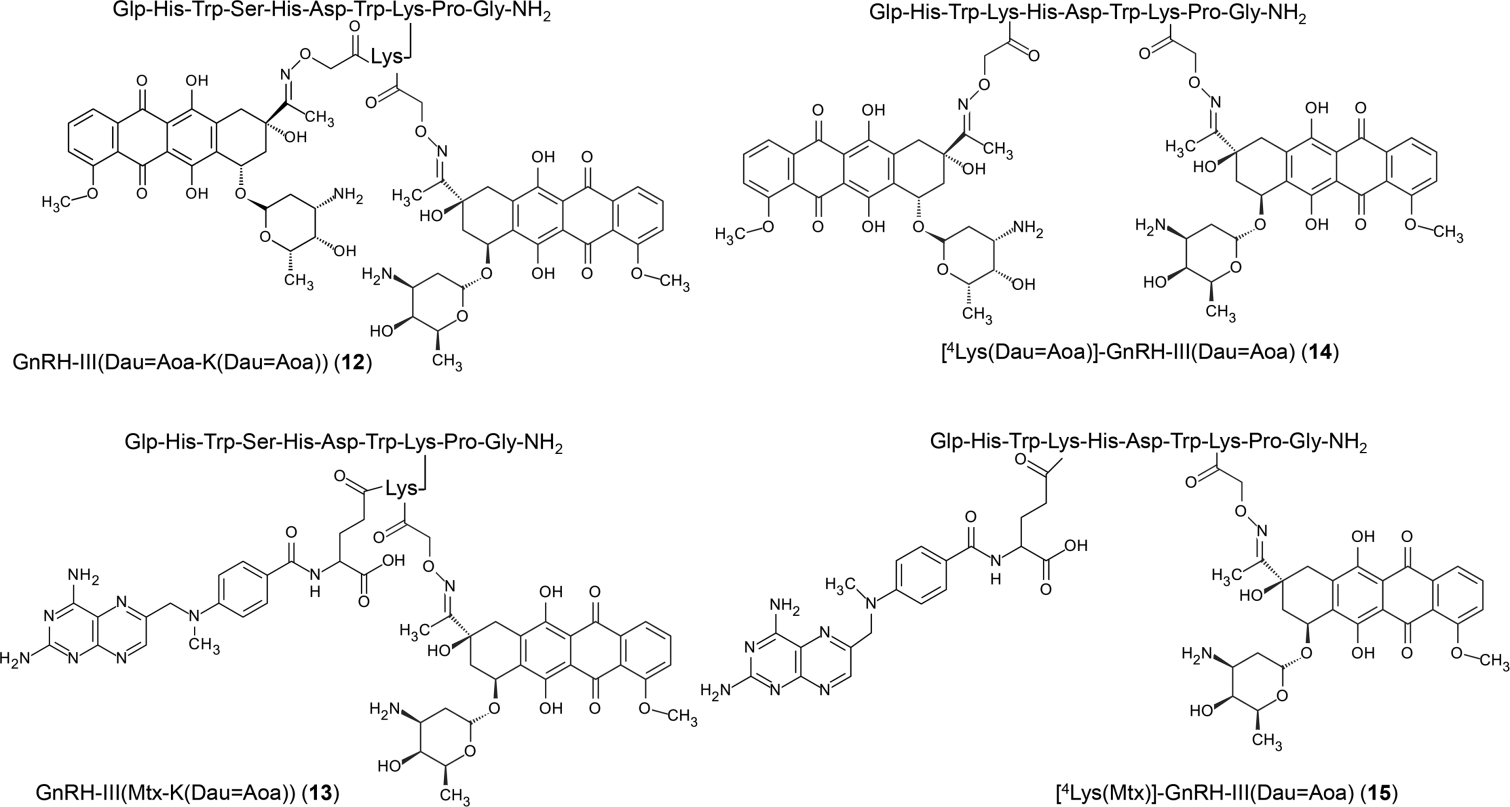
Chemical structures of the bifunctional GnRH-drug conjugates synthesized and investigated in the present work. Abbreviations: Aoa: aminooxyacetyl; Dox: doxorubicin; Dau: daunorubicin; Mtx: methotrexate; Glp: pyroglutamic acid.

In this study, the cardiotoxic effect of 15 GnRH-III–drug conjugates [14–15,20–25] as potential drug delivery systems was determined and compared with that of Zoptarelin doxorubicin (AN-152), a compound that reached phase III clinical trials. The codes of the GnRH–drug conjugates described here are shown in Figure 1 and Figure 2.

The GnRH derivatives were prepared by solid phase peptide synthesis (SPPS) according to Fmoc/*t*-Bu strategy (Supporting Information File 2). Three drug molecules, doxorubicin (Dox), daunorubicin (Dau) and methotrexate (Mtx), were employed in the preparation of drug conjugates. Dox has three potential conjugation sites; i) a primary OH group at C-14 on the aglycone part, which is suitable for ester bond formation, ii) an oxo group at C-13 is available for the generation of an oxime linkage and iii) the amino group on the daunosamine sugar moiety, which can be used for amide bond formation. The difference between Dox and Dau is the lack of the primary OH group in the case of the latter one. Therefore, Dau cannot be attached to peptide carriers via an ester bond. Mtx contains a glutamic acid whose carboxyl groups are suitable for the attachment to peptides through amide bond (this can be carried out on a solid support, prior to the cleavage of the peptide from the resin). In the conjugates used as reference compounds, Dox was coupled to the Lys in position 8 of GnRH-III through glutaric acid linked via an ester bond (*O*-glut) (**1**) (similarly to AN-152) or an amide bond through the sugar moiety (glut) (**2**) in solution. Furthermore, an oxime linkage was formed between Dox and an aminooxyacetyl moiety connected to the enzymatically cleavable tetrapeptide spacer GFLG (**3**). These conjugates were prepared with the aim of comparing the influence of the homing peptide and of the type of the linkage on the toxic effect. Dau was coupled to the GnRH-III derivatives via oxime linkage in all cases. The oxime ligation was carried out under slightly acidic (pH 5) conditions [26] and the drug was attached directly either to GnRH-III (**4**) or to its derivatives in which Ser in position 4 was replaced by Lys(Ac) (**5**) or *N*-MeSer (**6**), modifications that increased the enzymatic stability of the conjugates [20]. Furthermore, Dau was attached to GnRH-III through two different Cathepsin B enzymatic cleavable spacers GFLG (**7**) and YRRL (**8**). These conjugates were used to compare the influence of sequence modification, the presence or absence of a spacer as well as the type of spacers on toxicity. Disulfide dimers were also developed based on conjugates **4**, **6** and **8,** resulting in compounds **9**, **10** and **11**. Four conjugates with two identical (Dau) or different (Dau and Mtx) drug molecules were also prepared. When the native GnRH-III was used, an additional Lys was incorporated to the side chain of Lys in position 8. A Dau was attached to its ε-amino group and the second Dau (**12**) or the Mtx (**13**) was connected to the α-amino group. In conjugate **14,** the two Dau molecules were linked to two lysines in positions 4 and 8, while in conjugate **15** Dau was replaced by Mtx on the ^4^Lys. Using these compounds, the effect of the type and number of drug molecules could be compared. The isomers of Mtx conjugates (either α- or γ-carboxyl linked) were not separated, because our previous studies showed no significant differences in the biological activity of Mtx isomers in conjugated form [24].

Fmoc-protected Dox was reacted with glutaric anhydride in DMF. The prepared Fmoc-Dox-14-*O*-hemiglutarate was used for conjugation using PyBOP in the presence of NMM, followed by the removal of Fmoc group with 10% piperidine in DMF providing the conjugates AN-152 and **1**. It is worth mentioning that an O–N acyl shift (from the aglycone OH group to the daunosamine NH_2_ group ca. in 10–20%) was observed during the synthesis of conjugates containing an ester bond. This could be detected by the MS fragmentation of the conjugate between the aglycon part and sugar moiety resulted in fragments in which the peptide was attached either the aglycon OH group or the amino group of sugar moiety [23]. When unprotected Dox was used for the reaction with glutaric anhydride, the amino group was modified with glutaric acid followed by its attachment to the ε-amino group of ^8^Lys in GnRH-III. The conjugation was carried out in the presence of PyBOP/HOBt coupling agents resulting in conjugate **2**. For the preparation of the oxime linkage, a Boc protected aminooxyacetic acid was attached to the Lys side chain on solid phase prior to the cleavage of the peptide derivatives from the resin. The oxime bond formation was carried out in 0.2 M NaOAc solution at pH 5 for 12 hours. In contrast to the synthesis of the conjugates with ester or amide bond, the oxime bond formation was almost quantitatively. In the case of the conjugates with two different drug molecules or Lys(Ac) in position 4, orthogonal protecting schemes were applied during the SPPS. For the preparation of the disulfide dimers, an additional Cys was attached to the ε-amino group of ^8^Lys of the GnRH-III derivatives. First, Dau was linked to the aminooxyacetyl moiety, followed by the oxidation in 10 mM Tris buffer, pH 8/DMSO (1:1, v/v) solution.

The purity of the prepared conjugates presented in the original manuscripts or in their Supplementary Information was over 95% in all cases and there were no free drug molecules among the impurities. The summary of the characteristic data of the conjugates are presented in Supporting Information File 2 (Table S1).

### In vitro cytostasis

The in vitro cytostatic effect of GnRH-III–drug conjugates was determined on MCF-7 human breast and HT-29 human colon adenocarcinoma cells by 3-(4,5-dimethylthiazol-2-yl)-2,5-diphenyltetrazolium bromide (MTT) assay as it was described in the published articles [14–15,20–25]. All investigated bioconjugates exerted in vitro cytostatic effect with IC_50_ values in low µM range except the one (**2**) with amide bond between the drug and carrier molecule. The data are summarised below in Table 1. The data indicated, that conjugate **1** in which Dox linked via an ester bond to the homing peptide showed the highest antitumor activity on both cell lines. The conjugates with one Dau linked through oxime linkage showed similar potency on both types of cell. Small improvement of cytostatic effect could be detected when enzyme labile spacer was incorporated between the drug and GnRH peptide (conjugates **7**, **8**) compared with the basic conjugate (**4**). The enhancement of activity was also detected in case of dimers (**9**–**10**) compared to the monomers (**4**, **6**, **8**). The highest improvement in cytostatic effect was observed when Ser in position 4 of GnRH-III was replaced by Lys(Ac) (**5**), especially on HT-29 cells. However, the incorporation of *N*-MeSer (**6**) in this position lowered the efficacy. The conjugates with two drug molecules (either identical (**12**, **14**) or different (**13**, **15**)) belonged to the most efficient conjugates but no further significant improvement was detected to the conjugate **5** with one Dau.

### Measurements on human endothelial cells (HUVEC)

#### The effect of chemotherapeutic drugs (doxorubicin, daunorubicin, methotrexate)

**Figure 3:**
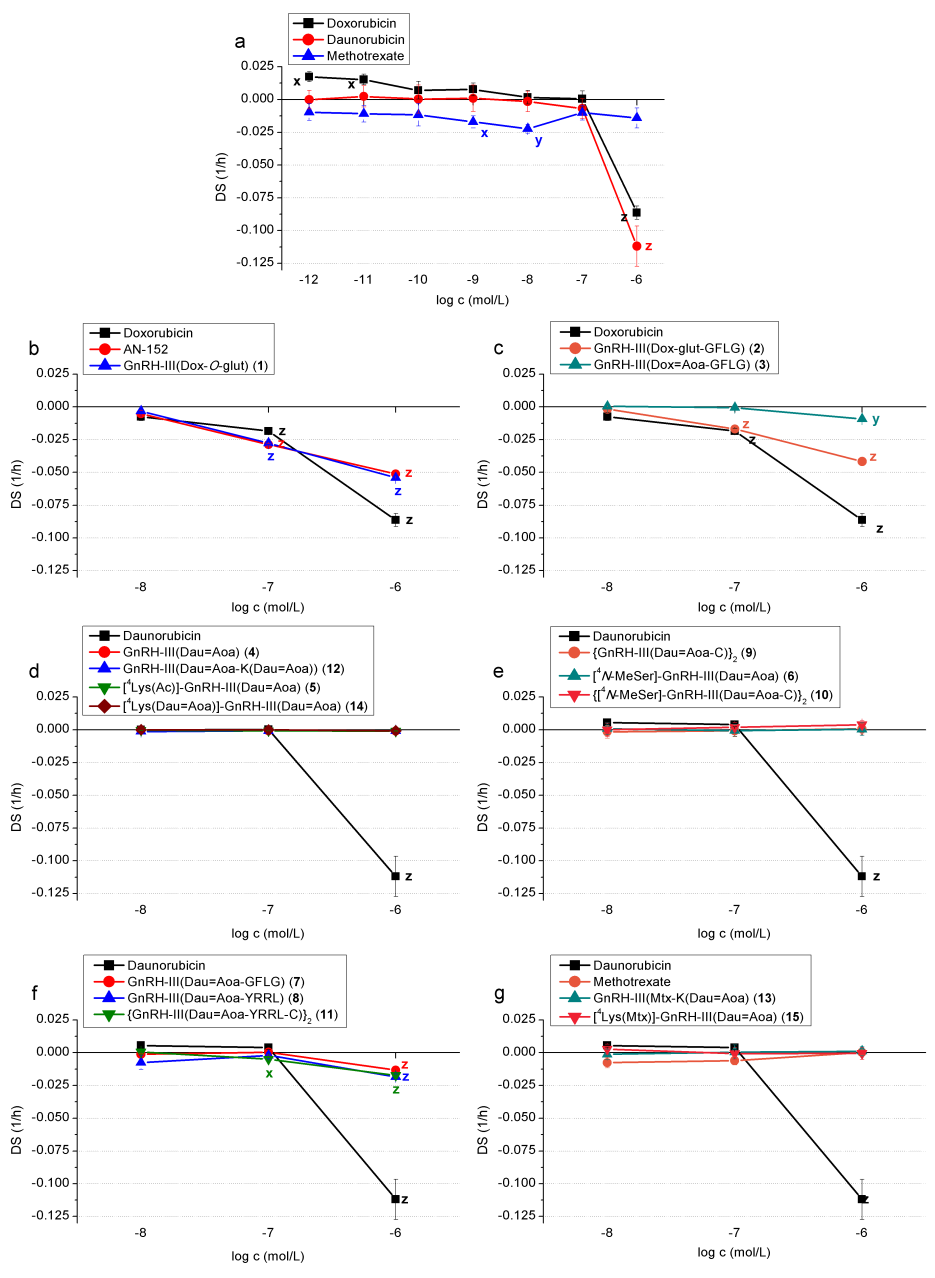
Long-term cytotoxic effects – impedimetrically registered negative effect on cell proliferation/viability – of chemotherapeutic drugs (doxorubicin, daunorubicin, methotrexate) and their GnRH-III conjugates on HUVEC cells. (a) Chemotherapeutic drugs (doxorubicin, daunorubicin, methotrexate); (b) GnRH conjugates containing doxorubicin without spacer sequence; (c) with spacer sequence GFLG; (d) oxime bond-linked, mono- and bifunctional daunorubicin-GnRH-III conjugates without spacer and modified in position 4 with Lys; (e) {GnRH-III(Dau=Aoa-C)}_2_ dimer and conjugates modified in position 4 with *N*-MeSer; (f) GFLG or YRRL spacer containing monomer and dimer conjugates (g) GnRH-III conjugates containing methotrexate and daunorubicin.

The effect of drugs used as chemotherapeutic agents was evaluated at seven different concentrations (10^−12^–10^−6^ mol/L) on human endothelial cells. Doxorubicin and daunorubicin significantly increased the impedance level (DS: 0.063 and 0.037 for doxorubicin and 0.047 and 0.055 for daunorubicin, respectively) at the two highest concentration levels (10^−6^ and 10^−7^ mol/L) on short-term treatment (Supporting Information File 1, Table S1). However, on long-term (0–72 hours) relation, a significant decrease was measured at 10^−6^ mol/L concentration for doxorubicin (DS: −0.086) and at 10^−6^ and 10^−7^ mol/L concentration for daunorubicin (DS: −0.019 and −0.007, respectively), indicating an eventual cytotoxic effect. Methotrexate showed effects on a much smaller scale – slight but significant decrease in impedance in the middle concentration range (10^−8^ and 10^−9^ mol/L) in long-term (DS −0.022 and −0.017, respectively, Figure 3a and Supporting Information File 1, Table S1a). Considering that in this experiment the major effects were found at the higher concentrations of drugs, in the following experiments with drug–peptide conjugates only the three highest concentrations (10^−8^, 10^−7^ and 10^−6^ mol/L) were investigated.

#### The effect of GnRH conjugates containing doxorubicin

In this study, three GnRH-III–Dox conjugates [GnRH-III(Dox-*O*-glut) (**1**), GnRH-III(Dox-glut-GFLG) (**2**), GnRH-III(Dox=Aoa-GFLG) (**3**)] were investigated in addition to AN-152, a doxorubicin-containing GnRH-I conjugate. In a short-term study, only conjugate **2** in which Dox is attached through an amide bond to the homing peptide showed a significant positive effect at 10^−6^ mol/L concentration (DS 0.058); nevertheless, the ester bond-linked conjugates AN-152 and **1** also proved to elicit a non-significant increase of the impedance measured (Supporting Information File 1, Table S2a). On long-term, at concentrations of 10^−7^ and 10^−6^ mol/L, besides doxorubicin (DS −0.019 and −0.046, respectively), AN-152 (DS −0.029 and −0.051, respectively), GnRH-III(Dox-*O*-glut) (DS −0.028 and −0.054, respectively) and GnRH-III(Dox-glut-GFLG) (DS −0.017 and −0.042) also showed a significant cytotoxic effect, while GnRH-III(Dox=Aoa-GFLG) had marginal effect (DS −0.009) only at the highest concentration level (10^−6^ mol/L) (Figure 3b and 3c; Supporting Information File 1, Table S2a).

#### The effect of oxime bond-linked daunorubicin–GnRH-III conjugates

Ten GnRH-III conjugates containing daunorubicin were investigated in this study. Seven of these compounds (GnRH-III(Dau=Aoa) (**4**), [^4^Lys(Ac)]-GnRH-III(Dau=Aoa) (**5**), [^4^*N*-MeSer]-GnRH-III (**6**), {GnRH-III(Dau=Aoa-C)}_2_ (**9**), {[^4^*N*-MeSer]-GnRH-III(Dau=Aoa-C)}_2_ (**10**), GnRH-III(Dau=Aoa)-K(Dau=Aoa)) (**12**) and [^4^Lys(Dau=Aoa)]-GnRH-III(Dau=Aoa) (**14**) completely lost the cytotoxic effect that was detected in the case of daunorubicin at high concentration levels, eliciting no effect at all. Even in short-term, only conjugate **10** increased significantly the measured impedance level (DS 0.017 in 10^−6^ mol/L), which is supposed to be a result of spreading of cells on the measuring electrode (Supporting Information File 1, Tables S3a and S4a). The three conjugates with spacer sequence GnRH-III(Dau=Aoa-GFLG) (**7**) (DS −0.013 in 10^−6^ mol/L), GnRH-III(Dau=Aoa-YRRL) (**8**) (DS −0.018 in 10^−6^ mol/L) and the dimeric {GnRH-III(Dau=Aoa-YRRL-C)}_2_ (**11**) (DS −0.005 in 10^−7^ mol/L and −0.017 in 10^−6^ mol/L) retained the significant cytotoxic effect of daunorubicin, although this effect was somewhat diminished. (Figure 3d–f,; Supporting Information File 1, Tables S3a and S4a).

#### The effect of GnRH-III conjugates containing methotrexate and daunorubicin

Two conjugates containing methotrexate and daunorubicin were examined (GnRH-III(Mtx-K(Dau=Aoa)) (**13**) and [^4^Lys(Mtx)]-GnRH-III(Dau=Aoa) (**15**)). On the short-term, conjugate **13** increased the measured impedance level significantly at the highest concentration (10^−6^ mol/L, DS 0.013) (Supporting Information File 1, Table S5a), while in the long-term study no significant effect was detected. Conjugate **15** showed no significant cytotoxic effect on either short- or long-term (Figure 3g; Supporting Information File 1, Table S5a).

### Measurements on human cardiomyocytes (HCM)

#### The effect of chemotherapeutic drugs (doxorubicin, daunorubicin, methotrexate)

**Figure 4:**
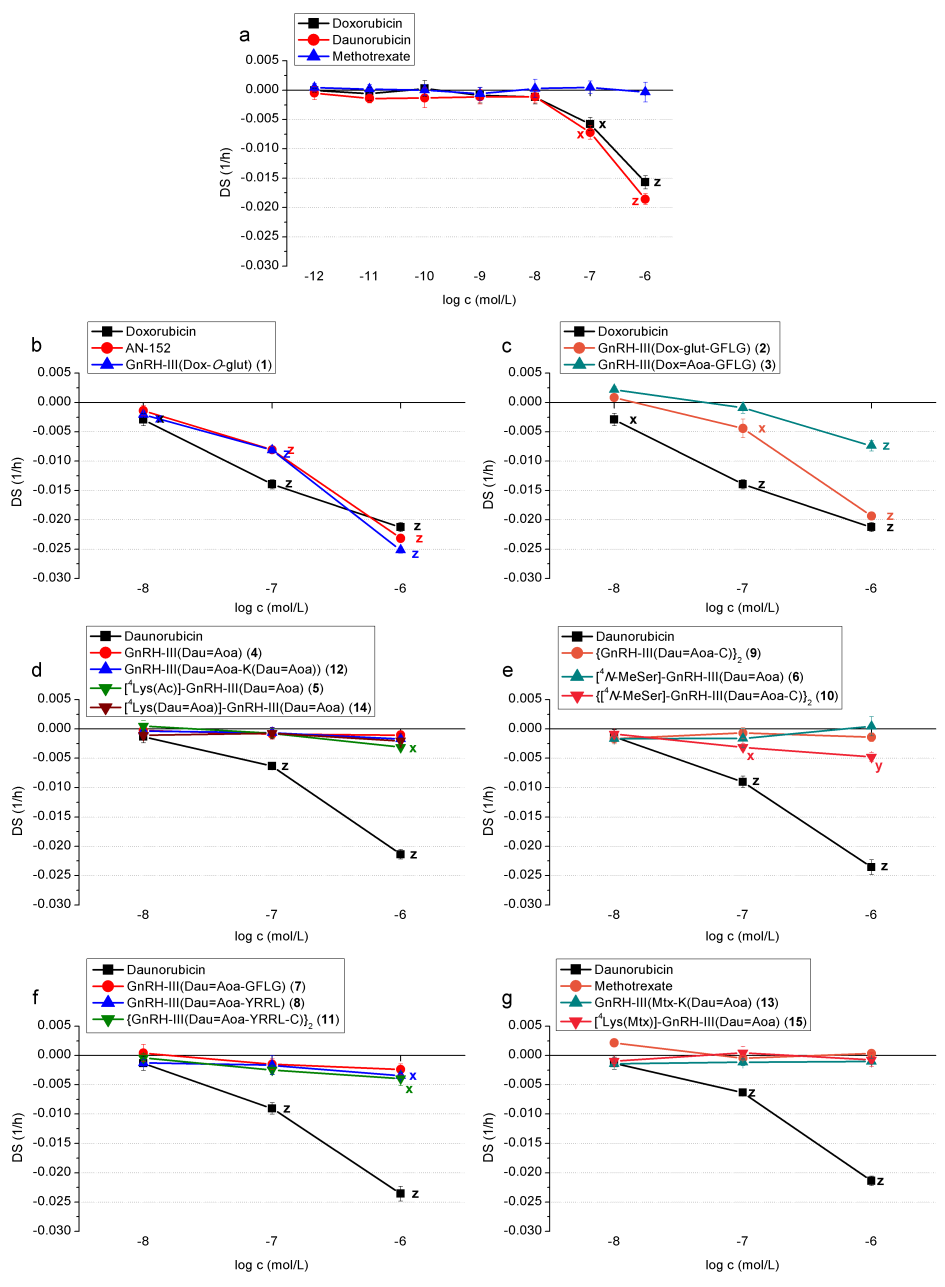
Long-term cytotoxic effects – impedimetrically registered negative effect on cell proliferation and viability – of chemotherapeutic drugs (doxorubicin, daunorubicin, methotrexate) and their GnRH-III conjugates on HCM cells. (a) Chemotherapeutic drugs (doxorubicin, daunorubicin, methotrexate); (b) GnRH conjugates containing doxorubicin without spacer sequence; (c) with spacer sequence GFLG; (d) oxime bond-linked, mono- and bifunctional daunorubicin-GnRH-III conjugates without spacer and modified in position 4 with Lys; (e) {GnRH-III(Dau=Aoa-C)}_2_ dimer and conjugates modified in position 4 with *N*-MeSer; (f) GFLG or YRRL spacer containing monomer and dimer conjugates; (g) GnRH-III conjugates containing methotrexate and daunorubicin.

Compared to their effect on endothelial cells, doxorubicin and daunorubicin showed quite a similar effect on cardiomyocytes. On short-term, both drugs increased the measured impedance level at the highest (10^−6^ mol/L) concentration (DS 0.019 and 0.031, respectively) (Supporting Information File 1, Table S1b), while on long-term they exhibited significant cytotoxic effect at the two highest concentration levels (10^−7^ and 10^−6^ mol/L) (DS −0.006 and −0.016 for doxorubicin, and −0.007 and −0.019 for daunorubicin, respectively) (Figure 4a). In case of methotrexate, however, no significant effect was measured (Figure 4a and Supporting Information File 1, Table S1b). As in the case of endothelial cells, in the following experiments, only the three highest concentrations (10^−8^, 10^−7^ and 10^−6^ mol/L) were evaluated. The investigated conjugates were the same as the ones used in the experiments with endothelial cells.

#### The effect of doxorubicin-containing GnRH conjugates

Two of the four investigated compounds, conjugate **1** (DS 0.012 in 10^−6^ mol/L) and **2** (DS 0.009 in 10^−8^ mol/L and 0.008 in 10^−6^ mol/L) exhibited a positive effect, similar to that of doxorubicin on short-term (the latter even at low concentration) (Supporting Information File 1, Table S2b). On long term, all of them had significant cytotoxic effect, conjugate **1** at all measured (10^−8^, 10^−7^ and 10^−6^ mol/L) concentrations (DS −0.002, −0.00811 and −0.025, respectively), AN-152 (DS −0.008 and −0.023, respectively) and conjugate **2** (DS −0.004 and −0.019, respectively) at 10^−7^ and 10^−6^ mol/L concentrations, while the cytotoxicity of conjugate **3** was expressed only at the highest (10^−6^ mol/L) concentration and with a reduced strength of efficiency (DS −0.007). (Figure 4b and 4c; Supporting Information File 1, Table S2b).

#### The effect of oxime bond-linked daunorubicin–GnRH-III conjugates

In short-term, only conjugate **9** increased the measured impedance level significantly at the highest (10^−6^ mol/L) concentration (DS 0.003) (Supporting Information File 1, Table S3b and S4b). On long-term, six conjugates (**4, 6, 7, 9, 12** and **14)** completely lost their cytotoxic effect. Conjugates **5, 8** and **11** had significant cytotoxic effect at a 10^−6^ mol/L concentration (DS −0.003, −0.004 and −0.004, respectively), while conjugate **10** at concentrations of 10^−7^ mol/L and 10^−6^ mol/L, too (DS −0.003 and −0.005, respectively). However, these cytotoxic effects were much weaker than that of daunorubicin (Figure 4d–f; Supporting Information File 1, Table S3b and Table S4b).

#### The effect of GnRH-III conjugates containing methotrexate and daunorubicin

In contrast to daunorubicin but similarly to methotrexate, none of the two investigated mixed drug–GnRH-III conjugates (**13** and **15**) exerted any significant cytotoxic effect on cardiomyocytes (Figure 4g; Supporting Information File 1, Table S5b).

## Discussion

The cardiovascular adverse effects of two chemotherapeutic agents, doxorubicin and daunorubicin, are well documented in the literature, while those of methotrexate are incidental or rare at most [18–19]. The results of our study are in agreement with the published ones: doxorubicin and daunorubicin had an in vitro cytotoxic effect both on cardiomyocytes and endothelial cells, while methotrexate had no cytotoxic effect on cardiomyocytes; furthermore, its cytotoxic effect was detectable only in short-term on endothelial cells, while the long-term negative effects disappeared. (Figure 3a and Figure 4a; Supporting Information File 1, Table S1a and b).

AN-152 is a GnRH-I conjugate containing doxorubicin, which has been described to have a strong antitumor effect on a large number of human cancers and reached phase III clinical trials; however, it has recently been discontinued [12]. This drug conjugate was used as a reference compound in our comparative study on the cardiotoxicity of GnRH-III conjugates. In these experiments, AN-152 exerted a cytotoxic effect (in 10^−6^ mol/L and 10^−7^ mol/L concentration) on cardiomyocytes, like that of free doxorubicin, but to a moderate extent. The same cytotoxic effect was observed on endothelial cells, as well; thus, despite its good antitumor effect, AN-152 does not represent a conjugate with the ideal safety profile. (Figure 3b and Figure 4b; Supporting Information File 1, Table S2a and b).

The GnRH-III analogue of AN-152, GnRH-III(Dox-*O*-glut) (**1**) showed comparable antitumor effect with that of AN-152 [14]. Results of our study indicate that conjugate **1** had similar cytotoxic effect as doxorubicin and AN-152 on endothelial cells at the two highest concentrations (10^−7^ and 10^−6^ mol/L). In contrast, these conjugates showed significantly lower toxicity at 10^−7^ mol/L, but a slightly higher toxicity at the highest concentration level (10^−6^ mol/L) on cardiomyocytes in a long-term experiment. It is worth mentioning that none of the compounds showed toxicity on any cell types in a short-term experiment. (Figure 3b and Figure 4b; Supporting Information File 1, Table 2a and b).

The two Dox–GnRH-III conjugates containing a GFLG spacer sequence (GnRH-III(Dox-glut-GFLG) (**2**), GnRH-III(Dox=Aoa-GFLG) (**3**)) retained the cytotoxic effect of doxorubicin both on HMC cardiomyocytes and HUVEC endothelial cells in the long-term study. The oxime bond-linked Dox conjugate (**3**) showed significantly lower toxicity on both cell types, while conjugate **2** had a similar concentration-dependent toxicity to that of the ester bond-linked conjugates on endothelial cells. The toxicity of the latter conjugate was lower on cardiomyocytes; it was significant at a concentration of 10^−7^ mol/L and moderate at 10^−6^ mol/L. (Figure 3c and Figure 4c; Supporting Information File 1, Table 2a and b). Interestingly, when Dox (conjugate **3**) was replaced by Dau (**7**) the toxicity on HUVEC cells was almost identical, while on HMC cells a lower effect was observed at the highest concentration level. The replacement of GFLG spacer to YRRL slightly but not significantly increased the toxicity of the conjugates, result that might be explained by the higher instability of YRRL spacer in cell culture medium. The lack of an enzymatically cleavable spacer resulted in conjugate **4** without toxic effects on any cardiac cell types. Because the oxime bond-linked Dau conjugates with or without spacers showed a similar antitumor effect on cancer cells, conjugate **4** could be the safest compound for cancer treatment. The conjugate **5** in which Ser in position 4 of GnRH-III was replaced by Lys(Ac) had a moderately increased toxicity on HMC cells at the highest concentration but not on HUVEC cells compared to **4**. In contrast, the incorporation of *N*-MeSer resulted in conjugate **6** that did not show toxicity on any cardiac cell types through the whole concentration range measured. These trends correlate with the antitumor activity of the conjugates. It was also found that the dimerization of Dau containing conjugates (**9**–**11**) did not lead to a significant change in toxicity on any cardiac cell types, except conjugate {[^4^*N*-MeSer]-GnRH-III(Dau=Aoa-C)}_2_ (**10**) that showed higher cytotoxicity on cardiomyocytes. It is worth mentioning that the dimerization increased the enzymatic stability of the conjugates without losing their antitumor effect. Because conjugate **9** with significant antitumor effect did not show cytotoxicity either on HMC or on HUVEC cells, it might be a promising and safe candidate for targeted therapy.

None of the conjugates (**12**–**15**) containing two drug molecules, independent on their type (Dau, Mtx) or attachment site, showed cytotoxicity on any of the tested cell types. The two Dau containing conjugates (**12**, **14**) showed a higher antitumor effect on MCF-7 human breast, HT-29 human colon and LNCaP human prostate cancer cells in vitro than that of conjugate **4**, but not of conjugate **5**. Conjugates **13** and **15** containing one Mtx and one Dau were more effective only on HT-29 cells in comparison with conjugate **4**. Considering the lack of toxicity of these conjugates with two drug molecules, they might be good candidates for tumor drug targeting for the treatment of GnRH receptor-positive cancers.

In respect of potential clinical applications of the tested conjugates in the future, a table was prepared (Table 1) which helps to compare cytotoxic efficacy (IC_50_) of the 15 GnRH-based antitumor conjugates in 3 reference tumor cell lines representing the most frequent malignancies (breast cancer – MCF-7, colorectal adenocarcinoma – HT-29 and acute monocytic leukemia – MonoMac6). In parallel, the cardiotoxic effects of the 15 conjugates are also demonstrated in the most important two targets of cardiac tissue (myocytes and endothelium). Data presented in Table 1 provide a good basis for structure–activity relationship analysis of the reported results. Comparison of IC_50_ values of conjugates possessing no cardiotoxicity (no cytotoxic effect both in HCM and HUVEC) shows that effectiveness of GnRH-based drug delivery is depending on the histological classification of the target tumor. Majority of the tested compounds (**4, 6, 12, 15**) possess the best IC_50_-s in the acute monocytic leukemia model (MonoMac6), while in some cases the breast cancer (**9**) or colorectal adenocarcinoma (**9, 13**) proved to be also sensible to the Dau containing conjugates. On the other hand there are even sad lessons of the comparative study, some mainly Dox containing conjugates (**1–3**) proved to have strong cardiotoxic effects; however, they had moderate cytotoxicity (IC_50_) in the compared three tumorous cell lines.

**Table 1:** Comparative study of cytotoxic effects and cardiotoxicity elicited by GnRH-based antitumor compounds (**1**–**15**) and reference substances (AN-152, Dox, Dau, Mtx). IC_50_ values show antitumor cytotoxic characteristics of the conjugates in human breast cancer (MCF-7), human colorectal adenocarcinoma (HT-29) and human acute monocytic leukemia (MM6) derived cell lines as representative tumor cells. Human cardiac myocytes (HCM) and human umbilical vein endothelial cells (HUVEC) as the chief targets of cardiotoxic compounds were studied and compared with tumor targets.

	Conjugates	IC_50_ values (μM)					Cardiotoxicity	
	
		MCF-7	HT-29	Ref.	MonoMac6	Ref.	HCM	HUVEC

	AN-152	0.2 ± 0.1	1.9 ± 0.3	[23]	0.035 ± 0.01	[27]	+++	+++
**1**	GnRH-III(Dox-*O*-glut)	0.1 ± 0.1	2.4 ± 0.2	[23]	0.039 ± 0.01	[27]	+++	+++
**2**	GnRH-III(Dox-glut-GFLG)	>100	>100	[14]	0.089 ± 0.02	[27]	+++	+++
**3**	GnRH-III(Dox=Aoa-GFLG)	5.4 ± 1.9	46.1 ± 6.1	[15]	0.430 ± 0.04	[28]	++	++
**4**	GnRH-III(Dau=Aoa)	6.5 ± 1.8	27.8 ± 4.2	[20]	>>1	[27]	0	0
**5**	[^4^Lys(Ac)]-GnRH-III(Dau=Aoa)	3.1 ± 1.7	7.4 ± 2.6	[20]	0.925 ± 0.07	[28]	+	0
**6**	[^4^*N*-MeSer]-GnRH-III(Dau=Aoa)	10.6 ± 2.1	nd	[20]	>>1	[28]	0	0
**7**	GnRH-III(Dau=Aoa-GFLG)	3.9 ± 1.2	22.5 ± 1.7	[21]	0.894 ± 0.25	[27]	+	++
**8**	GnRH-III(Dau=Aoa-YRRL)	1.8 ± 0.5	28.6 ± 5.5	[21]	>>1	[28]	+	++
**9**	[GnRH-III(Dau=Aoa-C)]_2_	4.1 ± 0.8	nd	[22]	1.688 ± 0.41	[27]	0	0
**10**	{[^4^*N*-MeSer]-GnRH-III(Dau=Aoa-C)}_2_	6.2 ± 1.5	nd	[22]	0.783 ± 0.09	[28]	+	0
**11**	{GnRH-III(Dau=Aoa-YRRL-C)}_2_	nd	nd		0.420 ± 0.05	[28]	+	++
**12**	GnRH-III(Dau=Aoa-K(Dau=Aoa))	3.0 ± 0.4	5.6 ± 2.0	[25]	>>1	[28]	0	0
**13**	GnRH-III(Mtx-K(Dau=Aoa))	5.4 ± 0.7	5.6 ± 3.0	[24]	1.413 ± 0.39	[28]	0	0
**14**	[^4^Lys(Dau=Aoa)]-GnRH-III(Dau=Aoa)	2.9 ± 0.9	6.8 ± 1.0	[25]	0.513 ± 0.04	[28]	+	0
**15**	[^4^Lys(Mtx)]-GnRH-III(Dau=Aoa)	5.8 ± 1.1	3.6 ± 1.5	[24]	>>1	[28]	0	0
	doxorubicin (Dox)	0.1 ± 0.0	0.1 ± 0.0	[23]	0.007 ± 0.0001	[27]	+++	+++
	daunorubicin (Dau)	0.4 ± 0.1	0.3 ± 0.2	[23]	0.00008 ± 0.0001	[27]	+++	+++
	methotrexate (Mtx)	nd	1.4 ± 0.6	[25]	0.008 ± 0.0002	[28]	0	+

## Conclusion

In the present study, the cytotoxic effect of sixteen GnRH-based conjugates containing doxorubicin, daunorubicin and methotrexate was determined on human cardiomyocytes and endothelial cells using an impedimetric technique. Seven conjugates with no cytotoxic effect on either cell types were identified, with two more conjugates with significantly milder cytotoxic effect. Based on these results, the monomeric GnRH-III(Dau=Aoa), the two multifunctional conjugates, [^4^Lys(Dau=Aoa)]-GnRH-III(Dau=Aoa) and GnRH-III(Dau=Aoa-K(Dau=Aoa)), the modified GnRH-III containing compound, ([^4^*N*-MeSer]-GnRH-III(Dau), the dimeric {GnRH-III(Dau-C)}_2_ and the two multifunctional conjugates containing two different chemotherapeutic drugs, GnRH-III(Mtx-K(Dau=Aoa)) and [^4^Lys(Mtx)]-GnRH-III(Dau=Aoa) are considered ideal compounds for tumor specific drug delivery with increased safety profiles; and in the future, could be the candidates for the development of effective chemotherapeutics with no cardiotoxic side effects.

## Experimental

For experimental procedures and structures of the GnRH-based conjugates investigated in the present study see Supporting Information File 2.

## Supporting Information

File 1 Experimental part – synthesis.

File 2 Supplementary tables – comparative data of short- and long-term effects of GnRH-drug conjugates on cardiomyocytes and endothelial cells.
