# Supplementary material for: Drug targeting to decrease cardiotoxicity – determination of the cytotoxic effect of GnRH-based conjugates containing doxorubicin, daunorubicin and methotrexate on human cardiomyocytes and endothelial cells
Source: Beilstein J Org Chem. 2018 Jun 28;14:1583–94. doi: 10.3762/bjoc.14.136 (PMC6037002; doi:10.3762/bjoc.14.136)
Supplement: File 1 — Experimental part – synthesis. [file Beilstein_J_Org_Chem-14-1583-s001.pdf]

Supporting Information File 1

for

Drug targeting to decrease cardiotoxicity – determination of the cytotoxic effect of GnRH-based conjugates containing doxorubicin, daunorubicin and methotrexate on human cardiomyocytes and endothelial cells

Livia Polgár[1,2], Eszter Lajkó[2], Pál Soós[1], Orsolya Láng[2], Marilena Manea[3], Béla Merkely[1], Gábor Mező[‡4,5], László Kőhidai[‡2 *]

Address: [1]Heart and Vascular Center, Semmelweis University, Városmajor u. 68., Budapest, 1122. Hungary, [2]Chemotaxis Research Group, Department of Genetics, Cell- and Immunobiology, Semmelweis University, Nagyvárad tér 4., Budapest, 1089. Hungary, [3]University of Konstanz, Department of Chemistry and Zukunftskolleg, Universitätsstrasse 10, 78467 Konstanz, Germany, [4]Eötvös Loránd University, Faculty of Science, Institute of Chemistry, Pázmány P. stny 1/A Budapest, 1117. Hungary and [5]MTA-ELTE Research Group of Peptide Chemistry, Pázmány P. stny 1/A Hungarian Academy of Science, Budapest, 1117. Hungary

[‡]Authors contributed equally to this work.

*Corresponding author:

Email: Kőhidai, László - kohlasz2@gmail.com

Experimental part – synthesis

Synthesis and characterization of peptides

The syntheses of various GnRH peptides and of the 16 investigated peptide-drug conjugates have previously been published and they are summarised below (Table S1).

Table S1: Chemical characterization of GnRH-III – drug conjugates.

**Compounds (code)**	**Bond**[a]	**HPLC (t_R)**	**ESI-MS (Mw_{calc})**	**ESI-MS (Mw_{av})**	**Ref.**
GnRH-III(Dox-*O*-glut) (**1**)	ester	30.2[2]	1893.0	1893.7	[1]
GnRH-III(Dox-glut-GFLG) (**2**)	amide	35.3[1]	2267.1	2266.8	[2]
GnRH-III(Dox=Aoa-GFLG) (**3**)	oxime	31.9[1]	2233.3	2232.9	[3]
GnRH-III(Dau=Aoa) (**4**)	oxime	29.1[1]	1841.9	1842.0	[4]
[^{4}Lys(Ac)]-GnRH-III(Dau=Aoa) (**5**)	oxime	29.9[1]	1856.0	1855.7	[5]
[^{4}*N*-MeSer]-GnRH-III(Dau=Aoa) (**6**)	oxime	29.3[1]	1925.1	1924.8	[5]
GnRH-III(Dau=Aoa-GFLG) (**7**)	oxime	29.6[1]	2215.3	2215.5	[4]
GnRH-III(Dau=Aoa-YRRL) (**8**)	oxime	27.5[1]	2429.1	2429.1	[4]
{GnRH-III(Dau=Aoa-C)}$_2$ (**9**)	oxime	27.8[2]	3888.1	3887.8	[6]
{[^{4}*N*-MeSer]-GnRH-III(Dau=Aoa-C)}$_2$ (**10**)	oxime	27.9[2]	3916.2	3916.0	[6]
{GnRH-III(Dau=Aoa-YRRL-C)}$_2$ (**11**)	oxime	24.5[2]	5064.3	5063.9	[4]
GnRH-III(Dau=Aoa-K(Dau=Aoa)) (**12**)	oxime<br>oxime	29.0[3]	2553.7	2553.5	[7]
GnRH-III(Mtx-K(Dau=Aoa)) (**13**)	amideo<br>xime	28.2[3]	2406.5	2406.1	[8]
[^{4}Lys(Dau=Aoa)]-GnRH-III(Dau=Aoa) (**14**)	oxime<br>oxime	30.3[3]	2465.6	2465.1	[7]
[^{4}Lys(Mtx)]-GnRH-III(Dau=Aoa) (**15**)	amide<br>oxime	28.2[3]	2319.0	2319.1	[8]

Abbreviations: GnRH-III: Glp-His-Trp-Ser-His-Asp-Trp-Lys-Pro-Gly-NH_2; Glp: pyroglutamic acid; gult: glutaric acid; Aoa: aminooxyacetyl; Dox: doxorubicin; Dau: daunorubicin; Mtx: methotrexate.

Analytical RP-HPLC was performed on:

[1]KNAUER 2501 HPLC system (KNAUER, Bad Homburg, Germany) using a Phenomenex Jupiter C18 column (250 mm x 4.6 mm) with 5 µm silica (300 Å pore size)

[2]SpectraSystem (Thermo Fisher Scientific, Dreieich, Germany) using a Hypersil GOLD C4 column (250 mm x 4.6 mm) with 5 µm silica (300Å pore size)

[3]UltiMate 3000 system (Dionex, Idstein, Germany) using a Vydac C18 column (250 mm x 4.6 mm) with 5 µm silica (300 Å pore size)

Linear gradient elution (0 min 0% B; 5 min 0% B; 50 min 90% B) with eluent A (0.1% TFA in water) and eluent B (0.1% TFA in acetonitrile : H_2O (80:20, v/v)) was used at a flow rate of 1 mL/min. Peaks were detected at 214 or 280 nm.

Electrospray (ESI)-mass spectrometric analyses were carried out on an Esquire 3000+ ion trap mass spectrometer (Bruker Daltonics, Bremen, Germany). Spectra were acquired in the 50 - 2500 *m/z* range. Samples were dissolved in a mixture of 50% methanol, 48% water and 2% acetic acid.

Synthesis of GnRH derivative–doxorubicin conjugates via ester linkage [1] and AN-152

GnRH derivative peptides were synthesized manually by solid phase peptide synthesis (SPPS) according to standard Fmoc/*t*-Bu strategy on a Rink-Amide MBHA resin. Peptides for conjugation via ester linkage were cleaved from the resin using a mixture of 95% TFA, 2.5% triisopropylsilane (TIS) and 2.5% water (v/v/v) for 2.5 h at rt. The peptides were precipitated with cold diethyl ether, washed three times with diethyl ether and solubilized in 100% acetic acid prior to freeze drying. The crude products were purified by semipreparative RP-HPLC and analyzed by mass spectrometry.

Then the syntheses of AN-152 and GnRH-III(Dox-*O*-glut) conjugates were carried out according to Nagy et al. [9]. Briefly, the daunosamine moiety of doxorubicin was first protected with Fmoc (by the reaction with Fmoc-OSu in DMF). After that, using glutaric anhydride, the Fmoc-Dox-14-*O*-hemiglutarate was formed. This Dox derivative was attached to the ε-amino group of Lys of either D-Lys^6-GnRH-I or GnRH-III after preactivating the carboxyl group with PyBOP in the presence of NMM. The last step of the synthesis was the removal of the Fmoc protecting group of daunosamine with 10% piperidine in DMF for 5 min at rt. Both bioconjugates, AN-152 and GnRH-III(Dox-*O*-glut) were purified by RP-HPLC and analyzed

by mass spectrometry. The overall yields of the syntheses were moderate 15-20% in which the critical steps were the conjugation of Fmoc-Dox-14-*O*-hemiglutarate to the petides and the Fmoc removal.

Synthesis of a GnRH-III derivative–doxorubicin conjugate containing an amide linkage [2]

For the synthesis of a branched GnRH derivative, a Lys with a selectively removable protecting group Mtt was built in the position 8 of the GnRH-III sequence. Mtt protecting group of the ε-amino function of ^{8}Lys was removed by 2% TFA in DCM (6 × 5 min). After a neutralization step with 10% DIEA/DMF for 4 × 1 min, the GFLG tetrapeptide spacer was built up step by step using the Fmoc protocol. After completion of the synthesis, the peptide was cleaved from the resin as described above. The peptide was purified and characterized before conjugation to Dox derivative.

Doxorubicin hydrochloride was modified with equiv amount of glutaric anhydride in DMF in the presence of equiv DIEA at rt for 1 h. The purified compound was pre-activated with PyBOP in the presence of NMM in DMF for 15 mins, then the GnRH-III derivative was added to the reaction mixture. The reaction mixture was worked up after 2 h and the conjugate was isolated by HPLC. The overall yield of the synthesis was 29% because of the easier conjugation (amide bond formation instead of ester linkage) and the lack of Fmoc removal step.

Synthesis of GnRH-III derivative–anthracycline conjugates via oxime linkage [3-5]

For the synthesis of GnRH-III derivatives for conjugation through oxime bond, a selectively removable Mtt amino protecting group was applied. When Ser was replaced by Lys(Ac) in position 4, the side chain of this Lys was protected with the Dde group. Prior to the Mtt cleavage, in this case, the Dde was removed with 2% hydrazine in DMF (2 x 15 min); subsequently, the acetylation of the ε-amino group was achieved by the treatment with Ac_2O/DIPEA/DMF (1:1:3, v/v/v) for 1 h. The Mtt protecting group of the ε-amino function of ^{8}Lys was removed by 2% TFA in DCM (6 × 5 min). After the specific modification of ^{8}Lys (built up the spacer sequences and/or attach Boc or bis-Boc-protected aminooxyacetic acid (Aoa)), the peptides were cleaved from the resin using a mixture of 95% TFA, 2.5% triisopropylsilane (TIS) and 2.5% water (v/v/v) for 2.5 h at rt. The peptides were precipitated with cold diethyl ether, washed three times with diethyl ether and solubilized in 100% acetic

acid prior to freeze drying. The crude products were purified by semipreparative RP-HPLC and analyzed by mass spectrometry.

The conjugation of daunorubicin or doxorubicin to aminooxyacetylated GnRH-III derivatives *via* an oxime bond was carried out in 0.2 M sodium or ammonium acetate buffer, pH = 5.0, at a peptide concentration of 10 mg/mL. The drug was added to a 20% excess compared to the aminooxyacetylated GnRH-III. The reaction mixture was stirred for 16 h at rt and then the conjugate was separated by semipreparative RP-HPLC. The purified oxime bond-linked drug – GnRH-III derivative conjugates were characterized by analytical RP-HPLC and mass spectrometry. The oxime bond formation run almost quantitatively. Therefore, the overall yields were 40–50% depending on the retention times of the conjugates and the free drugs. In some cases the separation of Dau from the conjugation might cause significant loss of conjugate to get Dau-free end product.

Synthesis of disulfide dimer of Dau–GnRH-III conjugates [6]

The synthesis of monomeric oxime bond-linked drug–GnRH-III derivative conjugates was carried out in the same way as described above, with the difference that an additional Cys was incorporated between the ε-amino group of ^{8}Lys and Aoa or the spacer sequence. The dimers were prepared from the monomeric conjugates by oxidation in 0.1 M Tris buffer (pH 8) – DMSO (1:1, v/v) mixture during an overnight reaction at rt, followed by the purification by semipreparative RP-HPLC. The purified dimer conjugates were characterized by analytical RP-HPLC and mass spectrometry. The oxidation step for dimerization run pretty well resulting in 60–80% yield (the overall yields are 30–40% in this way).

Synthesis of GnRH-III conjugates containing two Dau molecules [7]

To synthesize <EHWK(Aoa)HDWK(Aoa)PG-NH_2, the Mtt-protecting groups were removed from the ε-amino groups of ^{8}Lys and ^{4}Lys with 2% TFA in DCM (6 × 5 min) after completion of the synthesis of the protected decapeptides. After that, bis-Boc-Aoa-OH was attached to the free ε-amino groups after pre-activation with PyBOP in the presence of NMM (10 equiv to the resin capacity; coupling time 45 min). In case of <EHWSDWK(Aoa-K(Aoa))PG-NH_2, prior to the coupling of Aoa derivative, both Fmoc and Mtt protecting groups were removed from the additional Lys coupled to ^{8}Lys as described above. Aminooxyacetylated peptides were cleaved from the resin using a mixture of 95% TFA, 2.5% TIS, and 2.5% water (v/v/v) for 2.5 h at room temperature and then precipitated with ice-cold diethyl ether, washed three

times with diethyl ether and solubilized in 100% acetic acid prior to freeze drying. The crude products were purified by semipreparative RP-HPLC and analyzed by mass spectrometry.

The conjugation of daunorubicin to the aminooxyacetylated GnRH-III derivatives was carried out as it was described above. The overall yield of the synthesis of conjugates with two Dau was lower compared with the conjugates with one Dau, especially in case of the conjugate in which daunomycines were attached to the same Lys residue (34% and 27%).

Synthesis of GnRH-III–drug conjugates containing both daunorubicin and methotrexate [8]

In order to synthesize <EHWK(Mtx)DWK(Dau=Aoa)PG-NH_2, the Dde protecting group of the ε-amino function of ^{4}Lys was selectively removed using 2% hydrazine in DMF (2 × 15 min). Methotrexate (2.5 equiv) was coupled on the resin to the ε-amino group of the lysine residue in the presence of PyBOP:HOBt:NMM (1:1:2); coupling time added up to 60 min.

For the preparation of <EHWSDWK(Mtx-K(Dau=Aoa))PG-NH_2, an additional lysine residue was coupled to the ε-amino group of ^{8}Lys. Therefore, the Mtt protecting group of ^{8}Lys was selectively removed from the protected linear peptide on the resin with 2% TFA in DCM (6 × 5 min), followed by the coupling of Fmoc-Lys(Mtt)-OH according to the above-mentioned protocol. After removal of the Fmoc-protecting group of this additional lysine residue, Mtx was coupled to the α-amino group of the branched lysine residue as described above. After coupling of Mtx, the ε-amino groups of ^{8}Lys and of the branched Lys were aminooxyacetylated using bis-Boc-Aoa-OH (2.5 equiv), which was attached to the free ε-amino group after pre-activation with 2.5 equiv PyBOP in the presence of 5 equiv NMM (1 × 45 min).

The side chain modified peptides were cleaved from the resin using a mixture of 95% TFA, 2.5% TIS and 2.5% water (v/v/v) for 2.5 h at room temperature and then precipitated with ice-cold diethyl ether, washed three times with diethyl ether and solubilized in 100% acetic acid prior to freeze drying. The crude products were purified by semipreparative RP-HPLC and analyzed by mass spectrometry.

The conjugation of daunorubicin to the aminooxyacetylated GnRH-III(Mtx) derivatives was carried out in 0.2 M sodium acetate buffer (pH 5.0), at a peptide concentration of 10 mg/mL. Daunorubicin was used in 30% excess compared to the aminooxyacetylated GnRH-III(Mtx) derivatives. The reaction mixtures were stirred at room temperature for 24 h and then subjected to RP-HPLC purification. The purified conjugates were characterized by analytical RP-HPLC and mass spectrometry. The attachment of Mtx on solid phase did not provide

crude product with fairly good quality that decrease the overall yields below 25% in both cases.

References

1. Schlage, P.; Mezo, G.; Orban, E.; Bosze, S.; Manea, M. *J. Control Release* **2011,** *156,* 170-178. doi:10.1016/j.jconrel.2011.08.005
2. Mezo, G.; Manea, M.; Szabo, I.; Vincze, B.; Kovacs, M. *Curr. Med. Chem.* **2008,** *15,* 2366–2379. doi:10.2174/092986708785909157
3. Szabó, I.; Manea, M.; Orbán, E.; Csampai, A.; Bősze, S.; Szabó, R.; Tejeda, M.; Gaál, D.; Kapuvári, B.; Przybylski, M.; Hudecz, F.; Mező, G. *Bioconjugate Chem.* **2009,** *20,* 656–665. doi:10.1021/bc800542u
4. Orbán, E.; Mező, G.; Schlage, P.; Csík, G.; Kulic, Z.; Ansorge, P.; Fellinger, E.; Moller, H. M.; Manea, M. *Amino Acids* **2011,** *41,* 469–483. doi:10.1007/s00726-010-0766-1
5. Manea, M.; Leurs, U.; Orbán, E.; Baranyai, Z.; Ohlschlager, P.; Marquardt, A.; Schulcz, A.; Tejeda, M.; Kapuvári, B.; Továri, J.; Mező, G. *Bioconjugate Chem.* **2011,** *22,* 1320–1329. doi:10.1021/bc100547p
6. Schreier, V. N.; Mezo, G.; Orban, E.; Durr, C.; Marquardt, A.; Manea, M. *Bioorg. Med. Chem. Lett.* **2013,** *23,* 2145–2150. doi:10.1016/j.bmcl.2013.01.114
7. Leurs, U.; Mezo, G.; Orban, E.; Ohlschlager, P.; Marquardt, A.; Manea, M. *Biopolymers* **2012,** *98,* 1–10. doi:10.1002/bip.21640
8. Leurs, U.; Lajko, E.; Mezo, G.; Orban, E.; Ohlschlager, P.; Marquardt, A.; Kohidai, L.; Manea, M. *Eur. J. Med. Chem.* **2012,** *52,* 173–183. doi:10.1016/j.ejmech.2012.03.016
9. Nagy, A.; Schally, A. V.; Armatis, P.; Szepeshazi, K.; Halmos, G.; Kovacs, M.; Zarandi, M.; Groot, K.; Miyazaki, M.; Jungwirth, A.; Horvath, J. *Proc. Natl. Acad. Sci. U. S. A.* **1996,** *93,* 7269–7273. doi:10.1073/pnas.93.14.7269

Experimental part – Cellular samples

Cell cultures

In the present study, GnRH conjugates containing anti-tumour drugs (e.g., daunorubicin, doxorubicin) were investigated in order to evaluate whether the conjugates exert a decrease cardiotoxic effect compared to that of the free drugs. The cardiac myocytes and the vascular endothelium cells are the most frequently detected target cells of antitumor compounds and they are employed in our study. Human umbilical vein endothelial cells (HUVEC; Promocell, Biomedica, Wien, Austria) were cultured in Endothelial Cell Growth Medium with Endothelial Cell Growth Supplement Mix (Promocell, Biomedica, Wien, Austria) and 100 µg/mL penicillin/streptomycin. Primary human cardiac myocytes (HCM; Promocell, Biomedica, Wien, Austria) were cultured in Myocyte Growth Medium (Promocell, Biomedica, Wien, Austria).

Impedimetric measurements

The xCELLigence SP system was used for impedimetric measurements. The bottom of the wells of a 96-well E-plate is installed with a golden electrode array that constitutes the adhesion surface. Adhering cells – acting as insulators – change the electric impedance measured on the electrodes. The measured impedance change is in direct correlation with the number of adhering cells and the extent of adhesion. The results of impedance change are given in the form of Cell Index (CI) calculated by the xCELLigence system according to the following formula:

$$CI = \frac{Zt - Zb}{F}$$

where *Zt* is the measured impedance in a time point, *Zb* is the background impedance and *F* is an inbuilt factor (*F* = 15 at 10 kHz) depending on the frequency of the alternating current. The measurements took place in an incubator holding a 37 °C temperature and 5% carbon-dioxide atmosphere.

The experiments started with a measurement of baseline impedance for 1 hour. After that, the cells (10^4 cells/well) were added to the wells and allowed to adhere to the electrodes. When the adhering cells reached a balanced state in the wells, signified by the impedance curves reaching a plateau phase – roughly 20–28 hours for HMC and 24–26 hours for HUVEC – the free drugs at 10^{-12} – 10^{-6} M and the conjugates at 10^{-8} – 10^{-6} M final concentrations were

added to the wells. The changes in impedance were measured for at least 72 hours. For each measurement three parallels were measured.

The evaluation of responses followed the previously described strategy: acute effects of compounds were evaluated as a characteristic morphology of the curves in the 0–2 hours timeframe, while the 0–72 hours relation was considered as a benchmark of delayed, long-term cell physiological effects.

Statistical analysis

The evaluation of CI data was conducted by calculating the slope parameter with the integrated software RTCA 2.0 of the xCELLigence SP® system (Roche Applied Science, Indianapolis, IN, USA). The slope parameter is the rise of a straight line fitted to the measured data points in a chosen time period. This measurement method generates “dimensionless” data: the slope parameter equals the tangent of the angle between the straight line and the “*x*” axis. For quantification of cytotoxicity (or spreading/shrinking), delta slope (DS) value was used by calculating the difference between the slope of the treatment curve and that of the control curve. As the curves – after the treatments - showed a characteristic biphasic shape (a short, usually upsloping segment followed by a long, usually down sloping segment), DS values were calculated for these two phases (shown later as short-term and long-term effects). Slope values were normalized to control by calculating the difference between the slopes of examined and control curves.

RTCA 2.0 of the xCELLigence SP® was used to calculate and characterize IC50 values using the formula below:

$$Y = Bottom + (Top - Bottom)/(1 + 10^{\wedge}(Log\ IC_{50} - X))$$

The reported IC50 values of the text were calculated by using the same formula. In the case where not impedimetry but MTT assay was used to generate data of cytotoxicity (Table 1 MCF-7 and HT-29) there was no deviation between the two assays in respect of calculation of IC50 values.

For statistical analysis, one-way ANOVA test was applied. OriginPro 9 (Origin-Lab Corporation, Northampton, MA, USA) and RTCA Software 2.0 (Roche Applied Science, Indianapolis, IN, USA) were used for the analysis.
