# Supplementary material for: Drug targeting to decrease cardiotoxicity – determination of the cytotoxic effect of GnRH-based conjugates containing doxorubicin, daunorubicin and methotrexate on human cardiomyocytes and endothelial cells
Source: Beilstein J Org Chem. 2018 Jun 28;14:1583–94. doi: 10.3762/bjoc.14.136 (PMC6037002; doi:10.3762/bjoc.14.136)
Supplement: File 2 — Supplementary tables – comparative data of short- and long-term effects of GnRH-drug conjugates on cardiomyocytes and endothelial cells. [file Beilstein_J_Org_Chem-14-1583-s002.pdf]

Supporting Information File 2

for

Drug targeting to decrease cardiotoxicity – determination of the cytotoxic effect of GnRH-based conjugates containing doxorubicin, daunorubicin and methotrexate on human cardiomyocytes and endothelial cells

Livia Polgár[1,2], Eszter Lajkó[2], Pál Soós[1], Orsolya Láng[2], Marilena Manea[3], Béla Merkely[1], Gábor Mező[‡4,5], László Kőhidai[‡2 *]

Address: [1]Heart and Vascular Center, Semmelweis University, Városmajor u. 68., Budapest, 1122. Hungary, [2]Chemotaxis Research Group, Department of Genetics, Cell- and Immunobiology, Semmelweis University, Nagyvárad tér 4., Budapest, 1089. Hungary, [3]University of Konstanz, Department of Chemistry and Zukunftskolleg, Universitätsstrasse 10, 78467 Konstanz, Germany, [4]Eötvös Loránd University, Faculty of Science, Institute of Chemistry, Pázmány P. stny 1/A Budapest, 1117. Hungary and [5]MTA-ELTE Research Group of Peptide Chemistry, Pázmány P. stny 1/A Hungarian Academy of Science, Budapest, 1117. Hungary

[‡]Authors contributed equally to this work.

*Corresponding author:

Email: Kőhidai, László - kohlasz2@gmail.com

Supplementary tables – Comparative data of short- and long-term effects of GnRH-drug conjugates on cardiomyocytes and endothelial cells

Investigations of short- and long-term effects elicited by GnRH-drug conjugates provides information about diverse cell physiological responses induced by biologically active compounds. (i) The short-term effects are accomplished within several minutes or maximum 1–2 hours and they are mainly detected as acute responses (e.g., morphological changes like spreading or shrinking) of the cell. (ii) The long-term effects require more time to be detectable, the time required for development often depends on the cell cycle (24–72 hours). However, there is a long list of cell physiological responses induced in long-term relations, one of the most fundamental and significant response is cell proliferation. Therefore, in the present study we evaluate cytotoxic responses as chief characteristics of the long-term effects of GnRH-drug conjugates; nevertheless, provide comparative data of short- and long-term treatments as inducers of diverse cellular responses.

In the tables below, the results of impedimetric analysis on HUVEC and HCM cell lines are presented. The recorded data show spreading (short-term) and cytotoxic (long-term) responses. Statistically significant effects are highlighted in bold (one-way ANOVA test; * - $P < 0.05$; ** - $P < 0.01$; *** - $P < 0.05$).

Table S1: Short- and long-term cytotoxic effect of chemotherapeutic drugs (doxorubicin, daunorubicin, methotrexate) on (a) HUVEC and (b) HCM cell lines.

(a) HUVEC		10^{-12} mol/L	10^{-11} mol/L	10^{-10} mol/L	10^{-9} mol/L	10^{-8} mol/L	10^{-7} mol/L	10^{-6} mol/L
Short-term	Doxorubicin	0.008	**0.021***	-0.004	0.003	-0.008	**0.037*****	**0.063*****
	Daunorubicin	-0.005	0.008	-0.008	-0.008	0.018	**0.055****	**0.047****
	Methotrexate	**0.015***	0.013	0.012	**0.019***	0.013	0.002	0.001
Long-term	Doxorubicin	**0.017***	**0.015***	0.007	0.008	0.002	0.001	**-0.086*****
	Daunorubicin	-0.001	0.002	0.001	-0.00113	-0.001	**-0.007***	**-0.019*****
	Methotrexate	0.001	0.001	0.001	0.001	-0.001	-0.007	**-0.112*****
**(b) HCM**		**10^{-12} mol/L**	**10^{-11} mol/L**	**10^{-10} mol/L**	**10^{-9} mol/L**	**10^{-8} mol/L**	**10^{-7} mol/L**	**10^{-6} mol/L**
Short-term	Doxorubicin	0.001	0.002	0.002	-0.001	0.001	0.003	**0.019*****
	Daunorubicin	-0.001	-0.001	-0.001	-0.001	0.001	-0.001	**0.031*****
	Methotrexate	0.002	0.001	0.001	-0.001	0.001	0.001	0.005
Long-term	Doxorubicin	-0.001	-0.001	0.001	-0.001	-0.001	**-0.006***	**-0.016*****
	Daunorubicin	-0.001	-0.001	-0.001	-0.001	-0.001	**-0.007***	**-0.019*****
	Methotrexate	0.001	0.001	0.001	-0.001	0.001	0.001	-0.001

Table S2: Short- and long-term cytotoxic effect of GnRH conjugates containing doxorubicin on (a) HUVEC and (b) HCM cell lines.

(a) HUVEC		10^{-8} mol/L	10^{-7} mol/L	10^{-6} mol/L
Short-term	Doxorubicin	0.002	0.032	0.012
	An-152	-0.004	0.014	0.031
	GnRH-III(Dox-*O*-glut) (1)	-0.011	0.016	0.038
	GnRH-III(Dox-glut-GFLG) (2)	-0.004	0.016	**0.058****
	GnRH-III(Dox=Aoa-GFLG) (3)	-0.002	-0.003	-0.006
Long-term	Doxorubicin	-0.007	**-0.019*****	**-0.046*****
	An-152	-0.005	**-0.029*****	**-0.051*****
	GnRH-III(Dox-*O*-glut) (1)	-0.003	**-0.028*****	**-0.054*****
	GnRH-III(Dox-glut-GFLG) (2)	-0.002	**-0.017*****	**-0.042*****
	GnRH-III(Dox=Aoa-GFLG) (3)	0.0004	-0.001	**-0.009****
**(b) HCM**		**10^{-8} mol/L**	**10^{-7} mol/L**	**10^{-6} mol/L**
Short-term	Doxorubicin	0.001	-0.001	**0.057*****
	An-152	0.001	-0.001	0.008
	GnRH-III(Dox-*O*-glut) (1)	0.001	0.002	**0.012****
	GnRH-III(Dox-glut-GFLG) (2)	**0.009***	0.003	**0.008***
	GnRH-III(Dox=Aoa-GFLG) (3)	0.004	0.001	-0.002
Long-term	Doxorubicin	**-0.003***	**-0.014*****	**-0.021*****
	An-152	-0.001	**-0.008*****	**-0.023*****
	GnRH-III(Dox-*O*-glut) (1)	**-0.002***	**-0.008*****	**-0.025*****
	GnRH-III(Dox-glut-GFLG) (2)	0.001	**-0.004***	**-0.019*****
	GnRH-III(Dox=Aoa-GFLG) (3)	0.002	-0.001	**-0.007*****

Table S3: Short- and long-term cytotoxic effect of oxime bond-linked, bifunctional daunorubicin-GnRH-III conjugates on (a) HUVEC and (b) HCM cell lines.

	(a) HUVEC	10^{-8} mol/L	10^{-7} mol/L	10^{-6} mol/L
Short-term	**Daunorubicin**	-0.008	-0.002	0.003
	**GnRH-III(Dau=Aoa) (4)**	0.001	-0.003	-0.003
	**GnRH-III(Dau=Aoa-K(Dau=Aoa)) (12)**	0.009	0.010	-0.003
	**[4Lys(Ac)]-GnRH-III(Dau=Aoa) (5)**	-0.001	0.004	0.002
	**[4Lys(Dau=Aoa)]-GnRH-III(Dau=Aoa) (14)**	0.002	-0.005	0.006
Long-term	**Daunorubicin**	0.001	0.001	**-0.010*** **
	**GnRH-III(Dau=Aoa) (4)**	-0.001	-0.001	-0.001
	**GnRH-III(Dau=Aoa-K(Dau=Aoa)) (12)**	-0.001	-0.001	-0.001
	**[4Lys(Ac)]-GnRH-III(Dau=Aoa) (5)**	-0.001	-0.001	-0.001
	**[4Lys(Dau=Aoa)]-GnRH-III(Dau=Aoa) (14)**	0.001	-0.001	-0.001
	**(b) HCM**	**10^{-8} mol/L**	**10^{-7} mol/L**	**10^{-6} mol/L**
Short term	**Daunorubicin**	-0.001	-0.001	**0.028*** **
	**GnRH-III(Dau=Aoa) (4)**	-0.002	-0.001	0.001
	**GnRH-III(Dau=Aoa-K(Dau=Aoa)) (12)**	-0.001	-0.001	-0.001
	**[4Lys(Ac)]-GnRH-III(Dau=Aoa) (5)**	-0.002	-0.001	-0.005
	**[4Lys(Dau=Aoa)]-GnRH-III(Dau=Aoa) (14)**	0.001	-0.002	-0.001
Long term	**Daunorubicin**	**-0.001**	**-0.006*** **	**-0.021*** **
	**GnRH-III(Dau=Aoa) (4)**	-0.001	-0.001	-0.001
	**GnRH-III(Dau=Aoa-K(Dau=Aoa)) (12)**	-0.001	-0.001	-0.002
	**[4Lys(Ac)]-GnRH-III(Dau=Aoa) (5)**	0.001	-0.001	**-0.003***
	**[4Lys(Dau=Aoa)]-GnRH-III(Dau=Aoa) (14)**	-0.0011	-0.001	-0.002

Table S4: Short- and long-term cytotoxic effect of GnRH-III conjugates containing daunorubicin on (a) HUVEC and (b) HCM cell lines.

	(a) HUVEC	10^{-8} mol/L	10^{-7} mol/L	10^{-6} mol/L
Short-term	**Daunorubicin**	0.005	**0.016***	0.009
	**GnRH-III(Dau=Aoa-GFLG) (7)**	-0.002	-0.012	0.003
	**GnRH-III(Dau=Aoa-YRRL) (8)**	-0.010	-0.004	0.003
	**{GnRH-III(Dau=Aoa-YRRL-C)}$_2$ (11)**	0.022	0.002	0.014
	**{GnRH-III(Dau=Aoa-C)}$_2$ (9)**	0.001	0.008	0.014
	**[4*N*-MeSer]-GnRH-III(Dau=Aoa) (6)**	0.011	0.002	0.006
	**{[4*N*-MeSer]-GnRH-III(Dau=Aoa-C)}$_2$ (10)**	0.008	0.011	**0.017***
Long-term	**Daunorubicin**	0.006	0.004	**-0.058*** **
	**GnRH-III(Dau=Aoa-GFLG) (7)**	-0.001	0.001	**-0.013*** **
	**GnRH-III(Dau=Aoa-YRRL) (8)**	-0.007	-0.002	**-0.018*** **
	**{GnRH-III(Dau=Aoa-YRRL-C)}$_2$ (11)**	0.001	**-0.005***	**-0.017*** **
	**{GnRH-III(Dau=Aoa-C)}$_2$ (9)**	-0.002	-0.001	0.001
	**[4*N*-MeSer]-GnRH-III(Dau=Aoa) (6)**	0.001	-0.001	0.001
	**{[4*N*-MeSer]-GnRH-III(Dau=Aoa-C)}$_2$ (10)**	0.001	0.002	0.004

continued on next page

(b) HCM		10^{-8} mol/L	10^{-7} mol/L	10^{-6} mol/L
Short term	**Daunorubicin**	-0.001	**-0.009*** **	**0.030** **
	**GnRH-III(Dau=Aoa-GFLG) (7)**	-0.001	-0.001	0.003
	**GnRH-III(Dau=Aoa-YRRL) (8)**	-0.003	-0.001	-0.002
	**{GnRH-III(Dau=Aoa-YRRL-C)}$_2$ (11)**	-0.001	-0.001	-0.004
	**{GnRH-III(Dau=Aoa-C)}$_2$ (9)**	-0.001	0.002	**0.003***
	**[^{4}*N*-MeSer]-GnRH-III(Dau=Aoa) (6)**	-0.005	-0.001	0.001
	**{[^{4}*N*-MeSer]-GnRH-III(Dau=Aoa-C)}$_2$ (10)**	-0.002	-0.001	0.001
Long term	**Daunorubicin**	-0.001	**-0.009*** **	**-0.024*** **
	**GnRH-III(Dau=Aoa-GFLG) (7)**	0.001	-0.002	-0.002
	**GnRH-III(Dau=Aoa-YRRL) (8)**	-0.001	-0.002	**-0.004***
	**{GnRH-III(Dau=Aoa-YRRL-C)}$_2$ (11)**	-0.001	-0.002	**-0.004***
	**{GnRH-III(Dau=Aoa-C)}$_2$ (9)**	-0.002	-0.001	-0.001
	**[^{4}*N*-MeSer]-GnRH-III(Dau=Aoa) (6)**	-0.002	-0.002	0.001
	**{[^{4}*N*-MeSer]-GnRH-III(Dau=Aoa-C)}$_2$ (10)**	-0.001	**-0.003***	**-0.005** **

Table S5: Short- and long-term cytotoxic effect of GnRH-III conjugates containing methotrexate and daunorubicin on (a) HUVEC and (b) HCM cell lines.

(a) HUVEC		10^{-8} mol/L	10^{-7} mol/L	10^{-6} mol/L
Short-term	**Daunorubicin**	0.005	**0.016***	0.009
	**Methotrexate**	-0.003	0.003	**0.005** **
	**GnRH-III(Mtx-K(Dau=Aoa)) (13)**	0.001	0.002	**0.013***
	**[^{4}Lys(Mtx)]-GnRH-III(Dau=Aoa) (15)**	0.006	-0.002	0.006
Long-term	**Daunorubicin**	0.006	0.004	**-0.058*** **
	**Methotrexate**	-0.008	-0.006	0.001
	**GnRH-III(Mtx-K(Dau=Aoa)) (13)**	-0.001	0.001	0.001
	**[^{4}Lys(Mtx)]-GnRH-III(Dau=Aoa) (15)**	0.003	-0.001	-0.001
**(b) HCM**		**10^{-8} mol/L**	**10^{-7} mol/L**	**10^{-6} mol/L**
Short term	**Daunorubicin**	-0.001	-0.001	**0.028*** **
	**Methotrexate**	0.001	0.001	0.001
	**GnRH-III(Mtx-K(Dau=Aoa)) (13)**	-0.002	0.002	-0.003
	**[^{4}Lys(Mtx)]-GnRH-III(Dau=Aoa) (15)**	-0.003	-0.002	-0.001
Long term	**Daunorubicin**	-0.001	**-0.006*** **	**-0.021*** **
	**Methotrexate**	0.002	-0.001	0.001
	**GnRH-III(Mtx-K(Dau=Aoa)) (13)**	-0.001	-0.001	-0.001
	**[^{4}Lys(Mtx)]-GnRH-III(Dau=Aoa) (15)**	-0.001	0.001	-0.001
